# Evolutionary origins and genetic variation of the Seychelles treefrog, *Tachycnemis seychellensis* (Duméril and Bibron, 1841) (Amphibia: Anura: Hyperoliidae)

**DOI:** 10.1016/j.ympev.2014.02.004

**Published:** 2014-06

**Authors:** Simon T. Maddock, Julia J. Day, Ronald A. Nussbaum, Mark Wilkinson, David J. Gower

**Affiliations:** a Department of Genetics, Evolution and Environment, University College London, London WC1E 6BT, UK; b Department of Life Sciences, The Natural History Museum, London SW7 5BD, UK; c Museum of Zoology and Department of Ecology and Evolutionary Biology, University of Michigan, Ann Arbor, MI 48109-1079, USA

**Keywords:** DNA, Frogs, *Heterixalus*, Indian Ocean, Phylogeography

## Abstract

The hyperoliid frog *Tachycnemis seychellensis*, the only species of its genus, is endemic to the four largest granitic islands of the Seychelles archipelago and is reliant on freshwater bodies for reproduction. Its presence in the Seychelles is thought to be the product of a transoceanic dispersal, diverging from the genus *Heterixalus*, its closest living relative (currently endemic to Madagascar), between approximately 10–35 Ma. A previous study documented substantial intraspecific morphological variation among island populations and also among populations within the largest island (Mahé). To assess intraspecific genetic variation and to infer the closest living relative(s) of *T. seychellensis,* DNA sequence data were generated for three mitochondrial and four nuclear markers. These data support a sister-group relationship between *T. seychellensis* and *Heterixalus,* with the divergence between the two occurring between approximately 11–19 Ma based on *cytb p*-distances*.* Low levels of genetic variation were found among major mitochondrial haplotype clades of *T. seychellensis* (maximum 0.7% *p-*distance concatenated mtDNA), and samples from each of the islands (except La Digue) comprised multiple mitochondrial haplotype clades. Two nuclear genes (*rag1* and *tyr*) showed no variation, and the other two (*rho* and *pomc*) lacked any notable geographic structuring, counter to patterns observed within presumably more vagile Seychelles taxa such as lizards. The low levels of genetic variation and phylogeographic structure support an interpretation that there is a single but morphologically highly variable species of Seychelles treefrog. The contrasting genetic and morphological intraspecific variation may be attributable to relatively recent admixture during low sea-level stands, ecophenotypic plasticity, local adaptation to different environmental conditions, and/or current and previously small population sizes. Low genetic phylogeographic structure but substantial morphological variation is unusual within anurans.

## Highlights

• Although highly morphologically variable, the Seychelles treefrog *Tachycnemis seychellensis* lacks notable genetic phylogeographic structure.
• Samples from single islands (apart from La Digue) are not monophyletic.
• *Tachycnemis seychellensis* is supported as the sister taxon to a monophyletic *Heterixalus*.

## Graphical abstract

**Figure:**
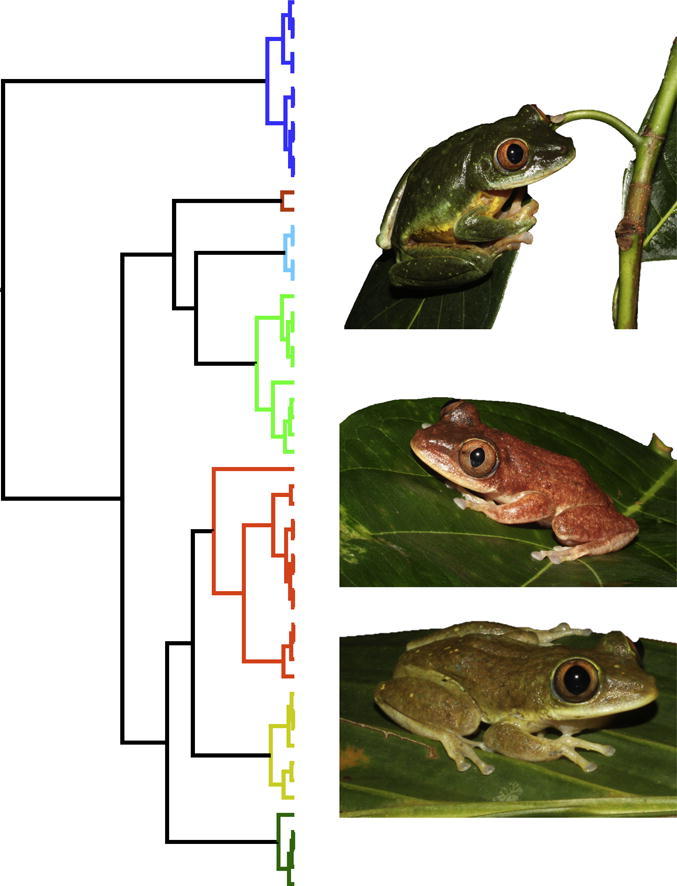

## 1 Introduction

Due to their isolation from potential confounding factors, remote islands have long been considered to provide important arenas for investigating evolution (Darwin, 1859). Most evolutionary studies of island biotas have focused on geologically recent volcanic island groups that have never been in contact with a large, ancient mainland, for example the Galápagos and Hawaii (e.g. Darwin, 1859; Gillespie, 2002). In contrast, the Seychelles archipelago (1600 km east of the nearest continental landmass) is formed of both granitic and coralline islands. The current granitic Seychelles are the remaining emergent part of a continental fragment, previously part of Gondwana, that was associated with India and Madagascar when they separated from Africa during the Cretaceous. At least some of the granitic Seychelles have always had some emergent land since the breakup of the Gondwanan supercontinent. Much of the continental Seychelles is currently submerged at an average depth of 55 m below sea level, forming the microcontinent ‘Seychellea’, comprising a total area of 129,650 km^2^ (Davies and Francis, 1964). During times of lowest stands in sea level (see Miller et al., 2005) all of the currently emergent granitic Seychelles would have been in contact. Fluctuations in sea level likely caused many episodes of dis- and reconnection among Seychelles islands, the most recent of which were within the last 10 ka (Colonna et al., 1996; Rohling et al., 1998; Siddall et al., 2003; Camoin et al., 2004; Miller et al., 2005). These fluctuations can be expected to have had a substantial impact on the amounts and spatial patterns of genetic variation of the resident biota.

It is unsurprising that most remote islands lack an endemic amphibian fauna given that the osmotic properties of amphibian skin (e.g. Balinsky, 1981; Duellman and Trueb, 1986) likely reduce their dispersal capabilities over saltwater substantially (Bossuyt and Milinkovitch, 2001; Darwin, 1859; Myers, 1953; Nussbaum, 1984). The Seychelles, however, has an amphibian fauna (∼12 species) comprising both frogs (Anura) and caecilians (Gymnophiona) that are restricted solely to the granitic islands (Nussbaum, 1984). Except for the widespread frog *Ptychadena mascareniensis* (Duméril and Bibron, 1841; Vences et al., 2004), all Seychelles amphibians are endemic. The endemic Seychelles frogs are confined to the four largest granitic islands of Mahé, Silhouette, Praslin and La Digue (Nussbaum and Wu, 1995, 2007; Taylor et al., 2012). Except for a preliminary study of sooglossid frogs (Taylor et al., 2012) molecular analyses have not yet been conducted to determine patterns of genetic variation among and within populations on different islands. Molecular techniques have been applied to several other Seychelles organisms, and substantial spatial structuring and deep genetic splits have been revealed, indicating the presence of cryptic lineages within several currently recognised lizard species (Rocha et al., 2010a,b, 2011, 2013; Valente et al., 2014), and a freshwater crab (Daniels, 2011).

Hyperoliidae is a pan-African family comprising >200 species in 17 or 18 genera (AmphibiaWeb, 2014; Frost, 2014) of small-medium sized treefrogs with representative species also found in the Seychelles and Madagascar (Vences et al., 2003a,b). The endemic Seychelles treefrog *Tachycnemis seychellensis*, the only species of its genus, is a sexually dimorphic, hyperoliid frog found on all four of the granitic islands of the Seychelles that support populations of frogs (Nussbaum and Wu, 1995). Like all hyperoliids *T. seychellensis* is an oviparous species with an aquatic larval stage, and it is restricted to areas close to water bodies (Nussbaum, 1984). The abundance and type of *T. seychellensis* habitat varies considerably across its range (Nussbaum and Wu, 1995). The southern islands of Mahé and Silhouette are higher (up to 905 and 750 m elevation, respectively), wetter and dominated by moist–wet forests, whereas the northern islands of Praslin (up to 367 m) and La Digue (333 m) are much lower and drier. Praslin has multiple rivers and streams, but La Digue lacks constant water sources at higher altitudes, and instead *T. seychellensis* is restricted here to marshy areas in the low-lying plateau on the west of this small island. The sizes of the four islands vary by more than an order of magnitude, ranging from 960 ha (La Digue) to 14,480 ha (Mahé), with Silhouette (1600 ha) and Praslin (4040 ha) somewhat intermediate.

Using univariate and multivariate analyses, Nussbaum and Wu (1995) discovered substantial external morphological variation among five populations of *T. seychellensis* from the four islands, including in adult body size and colouration, presence or absence of tubercles on various parts of the body and limbs, presence or absence of grooved digit discs, and several morphometric characters. Four morphometric characters, not dependant on sex, were found to vary significantly between all populations: internarial width, pes length, toe disc length, and length of metatarsal tubercle. An additional 10 male and two female characters varied significantly. Specimens from the more southerly islands of Mahé and Silhouette are morphologically the most similar to each other (Nussbaum and Wu, 1995). However, within Mahé (the only island for which more than one population was sampled), two populations of *T. seychellensis* (one marsh- and one stream-associated) only 1 km apart were as morphologically different from each other as they were to *T. seychellensis* on Silhouette. The populations of *T. seychellensis* on the more northerly islands (Praslin and La Digue) were morphometrically as distinct from each other as they were from the southern populations. Despite these large morphological differences, Nussbaum and Wu (1995) were impressed by (1) the fact that the four islands were likely connected as recently as 10 ka, (2) the intra-Mahé differences were as large as inter-island differences, (3) the substantial environmental differences across the four islands, and (4) the similar life history and bioacoustics of the different populations, and thus argued for the recognition of only a single species, one that has substantial and geographically structured morphological variation. Nussbaum & Wu’s single-species hypothesis for *T. seychellensis* could be challenged by high genetic diversity and/or substantial phylogeographic structure.

*Tachycnemis seychellensis* has a complicated taxonomic history. Since Dubois (1981) the species has been included in the monotypic genus *Tachycnemis* Fitzinger, 1843 and the species name has been attributed to Duméril and Bibron (1841) with Tschudi’s (1838) first use of the species name considered unavailable. Fitzinger (1843) established *Tachycnemis* only through bibliographic reference to its single included species (as described by Tschudi, 1838) without any explanation of his biological reasons (if any) for proposing the new genus. However, it has long been considered a phenotypically rather distinct hyperoliid (e.g. Günther, 1869), and Drewes (1984) hypothesised that it is the sister group of all other extant hyperoliids. More recently, based on analysis of concatenated mitochondrial DNA (mtDNA) and nuclear DNA (nuDNA), *T. seychellensis* has been inferred to be most closely related to the endemic Madagascan genus *Heterixalus* Laurent, 1944, which has 11 currently recognised species (Frost et al., 2006; Pyron and Wiens, 2011; Richards and Moore, 1996; Vences et al., 2003a,b; Wollenberg et al. 2007). However, although Wollenberg et al.’s (2007) main analysis of concatenated data recovered *Tachycnemis* and *Heterixalus* as sister taxa, five out of six trees inferred for the individual genes placed *Tachycnemis* within *Heterixalus*, although only *cox1* (mtDNA) and *rho* (nuDNA) did so with much support. Paraphyly of *Heterixalus* with respect to *Tachycnemis* was also found (though without strong support) by Vences et al. (2003b) in two mitochondrial gene trees. In contrast, these authors found that when three genes were concatenated, but using only two *Heterixalus* species, *Tachycnemis* and *Heterixalus* were sister taxa. Using multiple *Heterixalus* species in their analyses, Richards and Moore (1996), Frost et al. (2006), and Pyron and Wiens (2011) also recovered a *Tachycnemis*–*Heterixalus* sister-group relationship. Where trees for individual loci are discordant, coalescence-based methods can be expected to yield more accurate species phylogenies than multilocus concatenation (e.g. Edwards et al., 2007; Heled and Drummond, 2010; Kubatko and Degnan, 2007; Maddison and Knowles, 2006), but this latter approach has yet to be implemented in the case of *Tachycnemis* and *Heterixalus*.

Based on a sister-group relationship with *Heterixalus* and molecular dating analyses, the presence of *T. seychellensis* in the Seychelles is considered to originate from an overseas dispersal, with *Tachycnemis* diverging from its closest African/Madagascan relative an estimated 9.79–35.34 Ma (Crottini et al., 2012). This is in contrast to the sooglossid frogs that, as with the Seychelles caecilians, have probably been resident at least since Seychellea (the Seychelles microcontinent) was last part of Gondwana (Nussbaum, 1984).

Here we report phylogenetic analyses of mtDNA and nuDNA data (3228 base pairs (bp)) to (1) test the hypothesised sister-group relationship between *Heterixalus* and *Tachycnemis* and monophyly of the former genus, and (2) assess genetic variation within *T. seychellensis* across its range and test the hypothesis that it is a single, morphologically highly variable species.

## 2 Methods

### 2.1 Taxon sampling

*Tachycnemis seychellensis* tissue samples (liver, heart and muscle, frozen and stored at −80 °C) were obtained from 52 voucher specimens from the Seychelles islands of Mahé (15 samples), Silhouette (15 samples), Praslin (15 samples) and La Digue (7 samples) between 1988 and 1991; these correspond to four of the five populations sampled by Nussbaum and Wu (1995) (tissues of only a single Mahé population from Mare aux Cochons were available). Vouchers and tissues are deposited in the University of Michigan Museum of Zoology, USA (UMMZ) (see Appendix for details).

### 2.2 Laboratory protocols

Genomic DNA was extracted from liver, heart and muscle samples from the 52 *T. seychellensis* samples using the Qiagen DNeasy™ Tissue Kit. Three mitochondrial gene fragments were sequenced for all samples: cytochrome *b* (*cytb*), cytochrome oxidase subunit 1 (*cox1*) and 16S rRNA (*16s*). Four nuclear loci were also sequenced: rhodopsin exon 1 (*rho*), recombination activating gene 1 (*rag1*), tyronsinase precursor (*tyr*) and pro-opiomelanocortin (*pomc*). The *rag1* and *tyr* sequences showed no variation within *T. seychellensis,* and thus only a subset of individuals from each locality were included in the analyses of the relationships between *Tachycnemis* and *Heterixalus*.

Primer information is given in Table 1. Sequences were amplified using the polymerase chain reaction (PCR) with a total reaction volume of 15 μl: 1.5 μl of Bioline Buffer, 0.75 μl of MgCl2, 0.15 μl of dNTPs, 0.15 μl of Taq, 06 μl of both the forward and reverse primers, 0.6 μl of template DNA, and 10.65 μl ddH_2_O. Cycling conditions were: denature at 94 °C for 60s; followed by 35 (*16s*, *cytb*) or 40 (*cox1*, *tyr*, *pomc*, *rag1*) cycles of denaturing at 94 °C for 30 s, annealing at 48 °C (*cox1*), 50 °C (*16s*), 52 °C (*cytb*), 56 °C (*rag1*), 60 °C (*rho*), or 62 °C (*tyr*, *pomc*) for 30 s, and extending at 72 °C for 30 s; and a final extending step of 72 °C for 5 min.

### 2.3 Genetic variation within *T. seychellensis*

Sequences were proof-read using Sequencher v.4.8 and initially aligned using ClustalX v.2.0 (Larkin et al., 2007) using default settings before being checked by eye. All genes except the non-protein-coding *16s* were checked for pseudogenes and insertions by searching for stop codons and indels (e.g. Zhang and Hewitt, 1996) in MEGA5 (Tamura et al., 2011). The program DAMBE (Xia and Xie, 2001) was used to test for saturation using the test of Xia et al. (2003) across the different codon positions and the combined dataset.

To infer the phylogenetic relationships within *T. seychellensis* for the mitochondrial locus, we used Bayesian inference (BI) implemented in BEAST v.1.7.4 (Drummond et al., 2012). No outgroup taxa were used because BEAST estimates the position of the root in the tree assuming a molecular clock (Heled and Drummond, 2010). Input XML files were generated for BEAST analyses using BEAUti v.1.7.4. We selected best partitioning strategies and BEAST-compatible substitution models using PartionFinder (Lanfear et al., 2012).

The coalescent tree prior with exponential growth was used in BEAST based on the assumption that, after an initial colonisation, *T. seychellensis* likely expanded its range. Following the results of initial runs, uncorrelated relaxed clocks were rejected for all partitions and a strict clock implemented because constant rates could not be rejected. Two MCMC chains were run for 1 x 10^8^ generations for each partitioning strategy, with trees sampled every 10,000 generations to ensure convergence; this gave a total of 10,000 output trees per run. Convergence was checked by manual observation of the trace plots and ESS scores using Tracer v.1.5 (Rambaut and Drummond, 2009). All BEAST analyses were performed using the CIPRES Science Gateway v.3.1 (Miller et al., 2010).

To infer allelic phases from polymorphic sites in the nuDNA, the program PHASE v.2.1 (Stephens et al., 2001; Stephens and Scheet, 2005) was used, with input files created using seqPHASE (Flot, 2010). Haplotype networks under the median-joining algorithm (Bandelt et al., 1999) were produced to display intraspecific variation for *T. seychellensis* for the *pomc* and *rho* loci using the program NETWORK v.4.611 (fluxus-engineering.com).

Tajima’s *D* (Tajima, 1989) and Fu’s *Fs* (Fu, 1997) neutrality tests were used to investigate historical demographic properties in each island population of *T. seychellensis*. Negative values indicate a recent population expansion, values close to zero indicate a stable population, and any values considerably over zero indicate a recent population decrease. Both *D* and *Fs* were calculated using Arlequin v.3.5.1.3 (Excoffier and Lischer, 2010). Input files for Arlequin were created using PGDSpider v.2.0.3.0 (Lischer and Excoffier, 2012).

### 2.4 Testing monophyly of *Heterixalus*

The multispecies coalescent method as implemented in ^∗^BEAST (Heled and Drummond, 2010) was used to infer the species trees for *Tachycnemis* and *Heterixalus* spp., treating mtDNA (*cytb*, *cox1* and *16s*), *tyr*, *rag1* and *rho* as four separately evolving loci. Sequence data for *Heterixalus* spp. were previously published (Wollenberg et al., 2007) and obtained from GenBank, and those for *Tachycnemis* were newly generated. It is recommended to include a minimum of two specimens per species for ∗BEAST analyses so that there is a coalescent event with which to estimate population size (Heled and Drummond, 2010), but this was not possible for all species of *Heterixalus* because of inadequate specimen and/or character sampling in GenBank. For this reason *H. alboguttatus*, *H. boettgeri* and *H. carbonei* were excluded from these analyses. The remaining taxa nonetheless included representatives of all five *Heterixalus* species groups identified by Wollenberg et al. (2007).

Many studies using multilocus datasets do not partition by codon position, but we ran two sets of analyses in order to test for discrepancies between this *ad hoc* method and the optimal partitioning strategy identified by PartitionFinder (Table 3). Due to over parameterization, convergence was not reached in the identified optimal partitioning scheme in further analyses and therefore only the results of the locus partitioned analysis are used.

Preliminary analyses of the locus-partitioned dataset suggested that strict clocks be implemented for the mtDNA and *rhod* partitions and an uncorrelated lognormal relaxed clock for the *tyr* and *rag1*. Rates for molecular clocks were initially set at default (1.0) for all partitions and estimated relative to the mtDNA partition. Two MCMC chains were run for 2 × 10^8^ generations, with trees sampled every 10,000 generations, to ensure convergence was reached the first 5% were discarded as burn-in, although convergence was reached prior to this cut-off. The species-tree Yule-process prior was used with the piecewise linear and constant-root population-size model. Convergence of all parameters was verified using Tracer v.1.5 (Rambaut and Drummond, 2009).

## 3 Results

### 3.1 Monophyly of *Heterixalus*?

We aligned 1500 bp of mtDNA (consisting of 424 variable sites (v.s.), of which 391 were parsimony informative (p.s.)), 760 bp of *rag1* (62 v.s., 48 p.s.), 357 bp of *rho* (30 v.s., 15 p.s.), and 611 bp of *tyr* (56 v.s., 45 p.s.) giving a total sequence length of 3228 bp. Partitioning schemes and nucleotide models used in analyses are presented in Table 3.

**Fig. 1:**
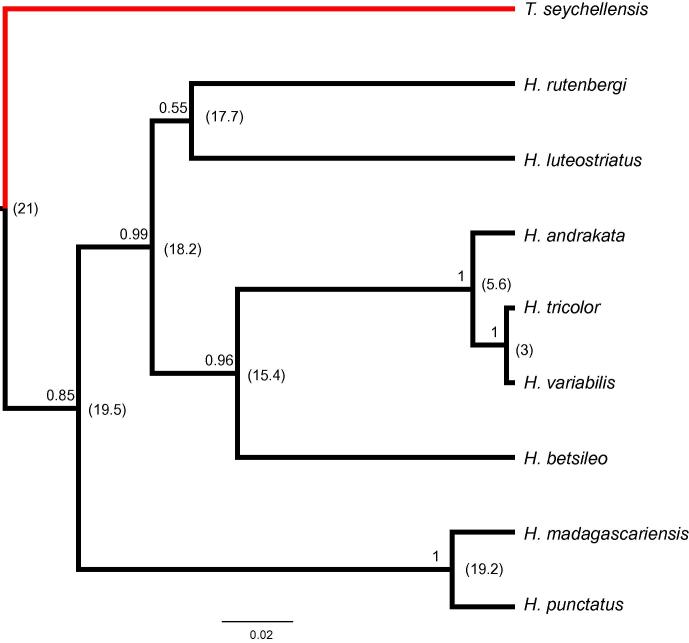
Bayesian species tree of the relationships between *Tachycnemis* and *Heterixalus* inferred using the multispecies coalescent in ∗BEAST. Numbers on branches are Bayesian posterior probabilities. The red branch indicates the placement of *T. seychellensis* whereas those of *Heterixalus* spp. are black. Numbers in parentheses at nodes are mean *p*-distances for *cytb* between two lineages. (For interpretation of the references to colour in this figure legend, the reader is referred to the web version of this article.)

Partitioning the ^∗^BEAST dataset by linked loci provided evidence for *T. seychellensis* being the sister taxon to a monophyletic *Heterixalus*; a sister-group relationship between *H. madagascariensis* and *H. punctatus*; for *H. andrakata* being the sister taxon to *H. tricolor* + *H. variabilis*; and for *H. betsileo* being the sister taxon to the *andrakata*–*tricolor*–*variabilis* clade (Fig. 1). The relationships of the remaining two species (*H. rutenbergi* and *H. luteostriatus*) are unresolved (Fig. 1).

Among *Heterixalus* species and clades, mean *p*-distances for *cytb* range from 3% to 19.5%, with the *p*-distance between *Tachycnemis* and *Heterixalus* being 21% (Fig. 1). Given an approximate rate of 0.6–1% per million years for *cytb* in amphibians (see Elmer et al., 2007), this marker indicates that *T. seychellensis* diverged from its closest sampled relative in the region of 11.5–19.2 Ma.

### 3.2 Genetic variation within *T. seychellensis*

We aligned three mitochondrial genes for the 52 specimens: *16s* consisted of 599 bp with three variable sites (v.s.), all of which were parsimony-informative (p.s.); *cytb* 763 bp (32 v.s., 30 p.s.); and *cox1* 786 bp (16 v.s., 13 p.s.). The dataset was almost complete, with very little missing sequence data across all genes and no genes missing for any individual. No saturation was detected. The *rag1* and *tyr* data were constant in 20 and 19 samples sampled across all populations, respectively. The *pomc* data consisted of 629 bp (7 v.s., 6 p.s.) for 50 specimens; and *rho* 337 bp (2 v.s., 2 p.s.) for 25 specimens. The sample size for *pomc* and *rho* was reduced because of a shorter amplified sequence length of some samples and because of a lack of confidence in the accuracy of the PHASE calling of the small number of variable sites.

**Fig. 2:**
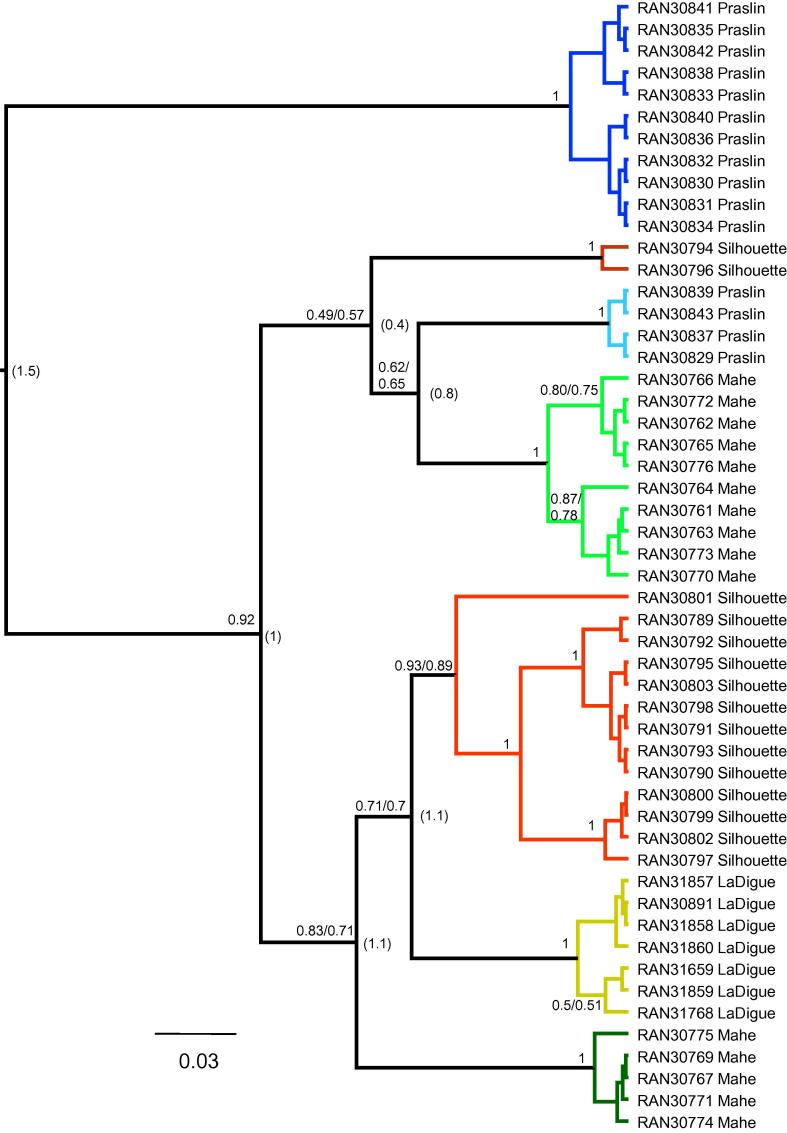
Bayesian inference tree for *Tachycnemis seychellensis* using three mtDNA gene fragments (*16s*, *cytb*, *cox1*) analysed with the BEAST software package. Numbers on branches are Bayesian posterior probabilities under AIC/BIC; when a single number is used both AIC and BIC schemes produced the same BPPs. Clade colours refer to those used in Fig. 3. Numbers in parentheses at nodes are mean *p*-distances for *cytb* between two lineages.

The best partitioning strategies and models as determined by PartitionFinder for the mtDNA analyses were the same under AIC and AICc but different under BIC (Table 2). Thus, two BEAST analyses were run under these alternatives, and the resulting tree topologies were identical and support values nearly so (Fig. 2).

**Fig. 3:**
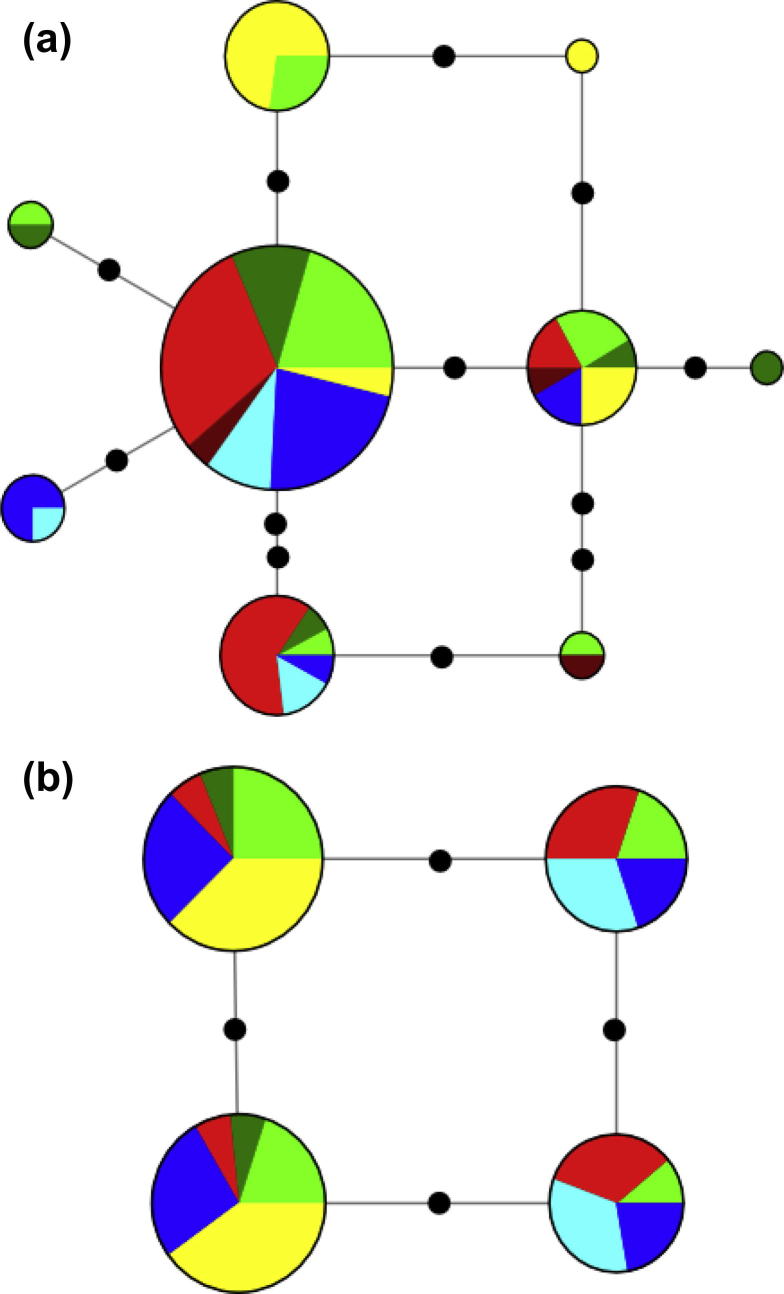
Median-joining haplotype networks for two nuDNA genes for *Tachycnemis seychellensis* determined using NETWORK: (a) *pomc*; (b) *rho*. Segment colours refer to clades in the mtDNA phylogenetic tree (Fig. 2). Black circles on connecting branches indicate the number of mutational steps between haplotypes.

The mtDNA has a maximum *p-*distance of 1.5% between any of the seven haplotype groups, and no simple geographic structure is observed in the mtDNA tree (Fig. 3), with samples from all islands except La Digue comprising two haplotype clades that are not sister groups, although not all relationships are well supported. The mean *p*-distance for *cytb* among the main mtDNA haplotype clades ranges from 0.4% to 1.5% (Fig. 2). Given an approximate rate of 0.6–1% per million years for *cytb* in amphibians (see Elmer et al., 2007), this marker suggests that extant mtDNA haplotype lineages of *T. seychellensis* began diverging approximately 0.75–1.25 Ma.

The two variable nuclear genes yielded networks with a general lack of geographic structure (Fig. 3). For *pomc* (Fig. 3a) there is a small amount of population structuring, with endemic haplotypes shared by multiple individuals within Praslin and Mahé. For *rho* (Fig. 3b) each of the four haplotypes are found on all islands except La Digue (two haplotypes).

Fu’s *Fs* results indicate recent expansions for all of the island populations with maximal significance (Table 4). Tajima’s *D* values suggest an opposite trend, with positive values indicating either a population size decrease or balancing selection, but Tajima’s *D* results are not significant for any island (Table 4).

## 4 Discussion

### 4.1 Monophyly of *Heterixalus*?

It has been estimated that *T. seychellensis* diverged from its closest living relative in Madagascar 9.79–35.34 Ma (Crottini et al., 2012), which implies transoceanic dispersal to the Seychelles given that this microcontinent split from Madagascar approximately 84 Ma (Ali and Aitchison, 2008; Plummer and Belle, 1995) and India by 64 Ma (McKenzie and Sclater, 1971; Norton and Sclater, 1979). Transoceanic dispersal remains a rarely documented phenomenon in amphibians, but see Hedges et al. (1992), Measey et al. (2007), Vences et al. (2003a,b, 2004).

Our analyses using the multispecies coalescent support previous studies based on concatenated multilocus DNA sequence data (e.g. Pyron and Wiens, 2011; Vences et al., 2003a,b; Wollenberg et al. 2007) that have hypothesised *T. seychellensis* to be the sister taxon to *Heterixalus*. Translation of *cytb p*-distances to divergence times among lineages produces estimates that fall within Crottini et al.’s (2012) estimate of 9.79–35.34 Ma for the divergence between *T. seychellensis* and its closest living relative. The *cytb p-*distances between *Tachycnemis* and *Heterixalus* spp. (Fig. 1) are clearly more in agreement with overseas dispersal than Seychelles-Africa or Seychelles-Madagascar Cretaceous vicariance as an explanation for the origin of *Tachycnemis* in the Seychelles.

### 4.2 Genetic variation within *T. seychellensis*

The results of our genetic analyses are consistent with Nussbaum and Wu’s (1995) interpretation that the Seychelles treefrog represents a single species. The low levels of genetic diversity within *T. seychellensis* and lack of notable phylogeographic structure can be explained by a rapid range expansion (supported by results for Fu’s *Fs*) and/or by multiple admixture events possibly during eustatic sea-level fluctuations. The latter is plausible given that all island populations, apart from La Digue, have multiple mtDNA haplotype clades and that nuDNA haplotypes show no clear geographic structuring. The relatively low levels of genetic variation within the Seychelles treefrog are comparable with several other Seychelles taxa such as *Drosophila* flies (Legrand et al., 2009) and freshwater turtles (Silva et al., 2010), although the turtles are probably a recent human introduction (Fritz et al., 2013). Conversely, studies of other taxa including lizards (Rocha et al., 2010a,b, 2011, 2013; Valente et al., 2014), a freshwater crab (Daniels, 2011), and a sooglossid frog (Taylor et al., 2012) have revealed much higher levels of inter-island genetic variation. It remains to be fully assessed whether differences in patterns of genetic variation among Seychelles organisms can be explained by ecology (and dispersal ability) and/or duration of residency. The presence of low genetic diversity and little phylogeographic structure but high morphological variation as is observed in *T. seychellensis* is unusual in (at least adult) anurans and it is difficult to find any examples in the literature (though see e.g., Gvoždík et al., 2008, 2010).

The combination of low levels of genetic diversity (and little phylogeographic structure) within *T. seychellensis* yet substantial morphological variation is perhaps best explained by rapid local adaptation to different environmental settings, ecophenotypic plasticity, or from previous genetic bottlenecks and/or continuing small population sizes (see also Nussbaum and Wu, 1995). The latter explanation seems unlikely on the islands of Mahé and Praslin where *T. seychellensis* is abundant in the sampled populations (STM, RAN, DJG *pers. obs.*). These explanations could be tested using population-genetic analyses of data from more rapidly evolving nuclear markers.

Genetic (*cytb p*-) distances between populations of *T. seychellensis* on different islands (see Fig. 2) provide no evidence for admixture between the islands after 200–333 ka. This might suggest that during the most recent sea-level fluctuations (∼10 ka), where all islands would have been connected (Colonna et al., 1996; Rohling et al., 1998; Siddall et al., 2003; Camoin et al., 2004; Miller et al., 2005), little migration occurred or, if migration did occur, mitochondrial haplotypes did not become fixed.

## 5 Conclusions

We find support for the sister-group relationship between *T. seychellensis* and a monophyletic *Heterixalus*. There is little genetic variation within *T. seychellensis*, even among populations on the four different islands within its range, and the variation is not strongly spatially structured. This is consistent with Nussbaum and Wu’s (1995) interpretation that there is a single species of Seychelles treefrog. The patterns of genetic variation that we have discovered do not allow us to reject Nussbaum and Wu’s (1995) proposal that substantial morphological variation within *T. seychellensis* is the result of local ecological adaptation and/or small population sizes now and/or in the past, though ecophenotypic plasticity might also be considered.

## Figures and Tables

**Table 1:** Primers used in this study for PCR and sequencing.

Gene fragment	Primer	Sequence (5′–3′)
*16s*	16SA-L a	CGCCTGTTTATCAAAAACAT
	16SB-H a	CCGGTCTGAACTCAGATCACGT
*cox1*	Amp-P3 F b	CAATACCAAACCCCCTTRTTYGTWTGATC
	Amp-P3 R b	GCTTCTCARATAATAAATATYAT
*cytb*	L14841 c	CTCCCAGCCCCATCCAACATCTCAGCATGATGAAACTTCG
	CB3H d	GGCAAATAGGAAGTATCATTCTG
*pomc*	POMC-1 e	GAATGTATYAAAGMMTGCAAGATGGWCCT
	POMC-2 e	TAYTGRCCCTTYTTGTGGGCRTT
*tyr*	Tyr1C f	GGCAGAGGAWCRTGCCAAGATGT
	Tyr1G f	TGCTGGGCRTCTCTCCARTCCCA
*rho*	Rhod1A^f^	ACCATGAACGGAACAGAAGGYCC
	Rhod1D^f^	GTAGCGAAGAARCCTTCAAMGTA
*rag1*	Amp-RAG1 F b	AGCTGCAGYCARTACCAYAARATGTA
	Amp-RAG1 R1 b	AACTCAGCTGCATTKCCAATRTCACA

a Palumbi et al. (1991).

b San Mauro et al. (2004).

c Kocher et al. (1989).

d Moritz et al. (1992).

e Wiens et al. (2005).

f Bossuyt and Milinkovitch (2000).

**Table 2:** Partitioning schemes and substitution models for the *Tachycnemis seychellensis* intraspecific mtDNA dataset. Numbers in parentheses refer to codon position.

	Partition scheme	Substitution models
AIC/AICc	*16s*	TrN
	*cytb* (1), *cox1* (1)	HKY
	*cytb* (2), *cox1* (2)	HKY
	*cytb* (3)	TrN
	*cox1* (3)	TrN + G

BIC	*16s*, *cytb* (1), *cox1* (1)	HKY
	*cytb* (2), *cox1* (2)	HKY
	*cytb* (3), *cox1* (3)	TrN + G

**Table 3:** Best-fit substitution models for partitions for the multispecies coalescent analysis.

	Partition	Substitution models
Locus partitions	*mtDNA*	GTR + G
	*rag1*	TrN + I
	*rho*	GTR + G
	*tyr*	SYM + I + G

**Table 4:** Population genetic statistics for Fu’s *Fs* and Tajima’s *D* for mtDNA data for 52 *Tachycnemis seychellensis*.

Island	*N*	*Fs*	*p*-Values	Tajima’s D	*p*-Values
Mahé	15	−8.99022	0.00000	1.59096	0.96300
Silhouette	15	−11.24523	0.00000	0.24311	0.62500
Praslin	15	−10.71708	0.00000	1.52485	0.95600
La Digue	7	−9.21700	0.00000	0.20619	0.65400
