# Supplementary material for: Evolutionary origins and genetic variation of the Seychelles treefrog, Tachycnemis seychellensis (Duméril and Bibron, 1841) (Amphibia: Anura: Hyperoliidae)
Source: Mol Phylogenet Evol. 2014 Jun;75(100):194–201. doi: 10.1016/j.ympev.2014.02.004 (PMC4101239; doi:10.1016/j.ympev.2014.02.004)
Supplement: Supplementary data 1 [file mmc1.docx]

**Appendix**

GenBank accession numbers and locality information for *Tachycnemis seychellensis* and *Heterixalus* *spp*. Island localities given only for Seychelles specimens (all *Heterixalus* are from Madagascar). *Heterixalus* data from previous studies; *Tachycnemis* data newly generated. Specimens with RAN prefix are from the tissue collection of the University of Michigan Museum of Zoology; other specimens are as reported by Wollenberg et al. (2007).

| **Species** | **Island** | **Specimen** | **mtDNA (*16s*, *cytb, cox1*)** | ***rag1*** | ***pomc*** | ***tyr*** | ***rho*** |
| --- | --- | --- | --- | --- | --- | --- | --- |
| *Tachycnemis seychellensis* | Mahé | RAN30761 | KJ551572, KJ551624, KJ551676 | KJ551817 | KJ551713 | KJ551805 |  |
| *T. seychellensis* | Mahé | RAN30762 | KJ551573, KJ551625, KJ551677 | KJ551818 | KJ551714 | KJ551806 |  |
| *T. seychellensis* | Mahé | RAN30763 | KJ551574, KJ551626, KJ551678 | KJ551819 | KJ551715 | KJ551807 | KJ551789 |
| *T. seychellensis* | Mahé | RAN30764 | KJ551575, KJ551627, KJ551679 | KJ551820 | KJ551716 |  | KJ551793 |
| *T. seychellensis* | Mahé | RAN30765 | KJ551576, KJ551628, KJ551680 | KJ551821 | KJ551717 |  | KJ551765 |
| *T. seychellensis* | Mahé | RAN30766 | KJ551571, KJ551623, KJ551675 |  | KJ551718 |  | KJ551788 |
| *T. seychellensis* | Mahé | RAN30767 | KJ551557, KJ551609, KJ551661 |  | KJ551719 |  | KJ551794 |
| *T. seychellensis* | Mahé | RAN30769 | KJ551558, KJ551610, KJ551662 |  | KJ551720 |  | KJ551795 |
| *T. seychellensis* | Mahé | RAN30770 | KJ551577, KJ551629, KJ551681 |  | KJ551721 |  |  |
| *T. seychellensis* | Mahé | RAN30771 | KJ551559, KJ551611, KJ551663 |  | KJ551722 |  |  |
| *T. seychellensis* | Mahé | RAN30772 | KJ551578, KJ551630, KJ551682 |  | KJ551723 |  | KJ551774 |
| *T. seychellensis* | Mahé | RAN30773 | KJ551579, KJ551631, KJ551683 |  | KJ551724 |  | KJ551791 |
| *T. seychellensis* | Mahé | RAN30774 | KJ551560, KJ551612, KJ551664 |  | KJ551725 |  | KJ551787 |
| *T. seychellensis* | Mahé | RAN30775 | KJ551561, KJ551613, KJ551665 |  | KJ551726 |  |  |
| *T. seychellensis* | Mahé | RAN30776 | KJ551580, KJ551632, KJ551684 |  | KJ551727 |  | KJ551786 |
| *T. seychellensis* | Silhouette | RAN30789 | KJ551581, KJ551633, KJ551685 | KJ551822 | KJ551728 | KJ551808 |  |
| *T. seychellensis* | Silhouette | RAN30790 | KJ551582, KJ551634, KJ551686 | KJ551823 | KJ551729 | KJ551809 | KJ551796 |
| *T. seychellensis* | Silhouette | RAN30791 | KJ551583, KJ551635, KJ551687 | KJ551824 | KJ551730 | KJ551810 | KJ551797 |
| *T. seychellensis* | Silhouette | RAN30792 | KJ551570, KJ551622, KJ551674 | KJ551825 | KJ551731 |  | KJ551798 |
| *T. seychellensis* | Silhouette | RAN30793 | KJ551569, KJ551621, KJ551673 | KJ551826 | KJ551732 |  | KJ551766 |
| *T. seychellensis* | Silhouette | RAN30794 | KJ551568, KJ551620, KJ551672 |  | KJ551733 |  | KJ551799 |
| *T. seychellensis* | Silhouette | RAN30795 | KJ551567, KJ551619, KJ551671 |  | KJ551734 |  | KJ551785 |
| *T. seychellensis* | Silhouette | RAN30796 | KJ551566, KJ551618, KJ551670 |  | KJ551735 |  | KJ551800 |
| *T. seychellensis* | Silhouette | RAN30797 | KJ551584, KJ551636, KJ551688 |  | KJ551736 |  | KJ551801 |
| *T. seychellensis* | Silhouette | RAN30798 | KJ551585, KJ551637, KJ551689 |  | KJ551737 |  | KJ551802 |
| *T. seychellensis* | Silhouette | RAN30799 | KJ551586, KJ551638, KJ551690 |  | KJ551738 |  |  |
| *T. seychellensis* | Silhouette | RAN30800 | KJ551587, KJ551639, KJ551691 |  | KJ551739 |  |  |
| *T. seychellensis* | Silhouette | RAN30801 | KJ551588, KJ551640, KJ551692 |  | KJ551740 |  | KJ551767 |
| *T. seychellensis* | Silhouette | RAN30802 | KJ551589, KJ551641, KJ551693 |  | KJ551741 |  | KJ551768 |
| *T. seychellensis* | Silhouette | RAN30803 | KJ551590, KJ551642, KJ551694 |  | KJ551742 |  | KJ551792 |
| *T. seychellensis* | Praslin | RAN30829 | KJ551565, KJ551617, KJ551669 |  | KJ551743 | KJ551811 | KJ551769 |
| *T. seychellensis* | Praslin | RAN30830 | KJ551598, KJ551650, KJ551702 | KJ551827 | KJ551744 | KJ551812 | KJ551770 |
| *T. seychellensis* | Praslin | RAN30831 | KJ551599, KJ551651, KJ551703 | KJ551828 | KJ551745 | KJ551813 | KJ551784 |
| *T. seychellensis* | Praslin | RAN30832 | KJ551600, KJ551652, KJ551704 | KJ551829 | KJ551746 |  | KJ551803 |
| *T. seychellensis* | Praslin | RAN30833 | KJ551601, KJ551653, KJ551705 | KJ551830 | KJ551747 |  | KJ551783 |
| *T. seychellensis* | Praslin | RAN30834 | KJ551602, KJ551654, KJ551706 | KJ551831 | KJ551748 |  | KJ551782 |
| *T. seychellensis* | Praslin | RAN30835 | KJ551603, KJ551655, KJ551707 |  | KJ551749 |  | KJ551771 |
| *T. seychellensis* | Praslin | RAN30836 | KJ551604, KJ551656, KJ551708 |  | KJ551750 |  | KJ551781 |
| *T. seychellensis* | Praslin | RAN30837 | KJ551591, KJ551643, KJ551695 |  | KJ551751 |  | KJ551772 |
| *T. seychellensis* | Praslin | RAN30838 | KJ551605, KJ551657, KJ551709 |  | KJ551752 |  |  |
| *T. seychellensis* | Praslin | RAN30839 | KJ551564, KJ551616, KJ551668 |  | KJ551753 |  | KJ551790 |
| *T. seychellensis* | Praslin | RAN30840 | KJ551606, KJ551658, KJ551710 |  | KJ551754 |  |  |
| *T. seychellensis* | Praslin | RAN30841 | KJ551607, KJ551659, KJ551711 |  | KJ551755 |  |  |
| *T. seychellensis* | Praslin | RAN30842 | KJ551608, KJ551660, KJ551712 |  | KJ551756 |  | KJ551804 |
| *T. seychellensis* | Praslin | RAN30843 | KJ551592, KJ551644, KJ551696 |  | KJ551757 |  | KJ551773 |
| *T. seychellensis* | La Digue | RAN30891 | KJ551593, KJ551645, KJ551697 | KJ551832 | KJ551758 | KJ551814 | KJ551780 |
| *T. seychellensis* | La Digue | RAN31659 | KJ551563, KJ551615, KJ551667 | KJ551833 | KJ551759 | KJ551815 | KJ551779 |
| *T. seychellensis* | La Digue | RAN31768 | KJ551594, KJ551646, KJ551698 | KJ551834 | KJ551760 | KJ551816 | KJ551778 |
| *T. seychellensis* | La Digue | RAN31857 | KJ551595, KJ551647, KJ551699 | KJ551835 | KJ551761 |  | KJ551777 |
| *T. seychellensis* | La Digue | RAN31858 | KJ551596, KJ551648, KJ551700 | KJ551836 | KJ551762 |  | KJ551775 |
| *T. seychellensis* | La Digue | RAN31859 | KJ551597, KJ551649, KJ551701 |  | KJ551763 |  | KJ551776 |
| *T. seychellensis* | La Digue | RAN31860 | KJ551562, KJ551614, KJ551666 |  | KJ551764 |  |  |
| *Heterixalus andrakata* |  | ZSM 508/2000 | EF646676, EF646609, - | EF646559 |  | EF646491 | EF646491 |
| *H. andrakata* |  | FGMV 2000.372 | EF646680, EF646613, - | EF646563 |  | EF646495 | EF646530 |
| *H. andrakata* |  | ZSM 566/2000 | EF646677, EF646610, - | EF646560 |  | EF646492 | EF646527 |
| *H. betsileo* |  | ZSM 356/2000 | EF646672, EF646605, - | EF646555 |  | EF646487 | EF646522 |
| *H. betsileo* |  | ZSM 682/2001 | EF646661, EF646594, - | EF646545 |  | EF646476 | EF646511 |
| *H. betsileo* |  | ZSM 718/2001 | EF646668, EF646601, - | EF646551 |  | EF646483 | EF646518 |
| *H. betsileo* |  | FGMV 2000.14 | EF646671, EF646604, - | EF646554 |  | EF646486 | EF646521 |
| *H. luteostriatus* |  | ZSM 697/2001 | EF646666, EF646599, - | EF646549 |  | EF646481 | EF646516 |
| *H. luteostriatus* |  | FGMV 2000.274 | EF646665, EF646598, - | EF646548 |  | EF646480 | EF646515 |
| *H. luteostriatus* |  | ZSM 426/2000 | EF646685, EF646618, - | EF646568 |  | EF646500 | EF646535 |
| *H. madagascariensis* |  | ZSM 569/2000 | EF646678, EF646611, - | EF646561 |  | EF646493 | EF646528 |
| *H. madagascariensis* |  | ZSM 568/2000 | EF646682, EF646615, - | EF646565 |  | EF646497 | EF646532 |
| *H. madagascariensis* |  | ZSM 684/2001 | EF646659, EF646592, - | EF646543 |  | EF646474 | EF646509 |
| *H. madagascariensis* |  | FGMV 2001.222 | EF646660, EF646593, - | EF646544 |  | EF646475 | EF646510 |
| *H. punctatus* |  | ZSM 683/2001 | EF646670, EF646603, - | EF646553 |  | EF646485 | EF646520 |
| *H. punctatus* |  | ZSM 349/2000 | EF646662, EF646595, - | EF646546 |  | EF646477 | EF646512 |
| *H. punctatus* |  | ZSM 571/2000 | EF646679, EF646612, - | EF646562 |  | EF646494 | EF646529 |
| *H. punctatus* |  | FGMV 2000.374 | EF646681, EF646614, - | EF646564 |  | EF646496 | EF646531 |
| *H. punctatus* |  | ZSM 572/2000 | EF646683, EF646616, - | EF646566 |  | EF646499 | EF646533 |
| *H. rutenbergi* |  | ZSM 361/2000 | EF646673, EF646606, - | EF646556 |  | EF646488 | EF646523 |
| *H. rutenbergi* |  | ZSM 789/2001 | EF646667, EF646600, - | EF646550 |  | EF646482 |  |
| *H. tricolor* |  | ZSM 700/2001 | EF646664, EF646597, - | EF646547 |  | EF646479 | EF646514 |
| *H. tricolor* |  | ZSM 463/2000 | EF646674, EF646607, - | EF646557 |  | EF646489 | EF646524 |
| *H. tricolor* |  | FGMV 2000.235 | EF646675, EF646608, - | EF646558 |  | EF646490 | EF646525 |
| *H. variabilis* |  | ZSM 425/2000 | EF646687, EF646620, - | EF646570 |  | EF646502 | EF646537 |
| *H. variabilis* |  | ZSM 608/2001 | EF646669, EF646602, - | EF646552 |  | EF646484 | EF646519 |
| *H. variabilis* |  | FGMV 2000.188 | EF646688, EF646621, - | EF646571 |  | EF646503 | EF646538 |
| *H. variabilis* |  | FGMV 2000.185 | EF646686, EF646619, - | EF646569 |  | EF646501 | EF646536 |
